# ERGA-BGE reference genome of *Diadema setosum:* the Black Longspine Urchin invading the Mediterranean sea

**DOI:** 10.12688/openreseurope.20681.1

**Published:** 2026-03-12

**Authors:** Katerina Vasileiadou, Tereza Manousaki, Astrid Böhne, Rosa Fernández, Nuria Escudero, Genoscope Sequencing Team, Alice Moussy, Corinne Cruaud, Karine Labadie, Lola Demirdjian, Benjamin Istace, Arnaud Couloux, Patrick Wincker, Pedro H. Oliveira, Jean-Marc Aury, Rita Monteiro

**Affiliations:** 1 Institute of Oceanography, Hellenic Centre for Marine Research, Heraklion, Creete, 70014, Greece; 2 Institute of Marine Biology Biotechnology and Aquaculture, Hellenic Centre for Marine Research, Heraklion, Crete, 70014, Greece; 3 Leibniz Institute for the Analysis of Biodiversity Change, Museum Koenig Bonn, Adenauerallee 127, Bonn, 53113, Germany; 4 Metazoa Phylogenomics Lab, Institute for Evolutionary Biology (CSIC-UPF). Passeig marítim de la Barceloneta 37-49. 08003, Barcelona, Spain; 5 Genoscope, Institut François Jacob, CEA, CNRS, Univ Evry, Université Paris-Saclay, Evry, 91057, France; 6 Génomique Métabolique, Genoscope, Institut François Jacob, CEA, CNRS, Univ Evry, Université Paris-Saclay, Evry, 91057, France

**Keywords:** Diadema setosum, genome assembly, European Reference Genome Atlas, Biodiversity Genomics Europe, Earth Biogenome Project, Diadematidae, Black Longspine Urchin

## Abstract

The *Diadema setosum* reference genome is important for understanding the species adaptation to the Mediterranean marine environment, where it has been newly introduced. The species is a Lessepsian migrant, gradually expanding to the eastern Mediterranean basin, driven by rising water temperature. *Diadema setosum* often dominates over native sea urchin species or coexists with them in rocky habitats.

Sea urchins are environment-forming species, as they are intensive grazers responsible for habitat degradation and bottom erosion. A high-quality reference genome could provide valuable insights into the adaptive ability of *D. setosum* populations, supporting better monitoring and conservation efforts. Additionally, the genome will also contribute towards having a record of recently introduced populations to the Mediterranean, allowing researchers to track their evolution over time.

A total of 22 contiguous chromosomal pseudomolecules were assembled from the genome sequence. This chromosome-level assembly encompasses 0.91 Gb, composed of 745 contigs and 101 scaffolds, with contig and scaffold N50 values of 2.2 Mb and 39.8 Mb, respectively.

## Introduction

*Diadema setosum* or Μακράκανθος Αχινός in Greek *,* also known as the Black Longspine Urchin, belongs to the Diadematidae family. The phylogeny of the *Diadema* species has shown that *D. setosum* is more divergent than the other *Diadema* species, forming two distinct clades: Clade A, which expands into the Indo-Pacific and Clade B, which is restricted to the Persian Gulf and Red Sea ( Lessios, 2000). Thus far, *D. setosum* spreading in the Mediterranean has been found to belong to the second clade ( Bronstein et al., 2017). The first record of the species was in 2006 from the coasts of Turkey. Since then, its distribution has expanded to the north-eastern Mediterranean coasts with the last references reporting occurrences up to the south Ionian Sea and the Sea of Marmara, with the abundances observed to increase dramatically since 2018 ( Zirler et al., 2023). Thermophilia of the Longspine Urchin favors the organism against the indigenous species, as the water temperature is rising in the Mediterranean due to the shifting of climatic conditions.

Sea urchins are considered habitat-forming species as they are intense algal grazers, which can cause the complete collapse of entire systems. *Diadema setosum* can alter benthic communities by regulating algal growth, which can lead to shifts in larval settlement and development ( Zirler et al., 2023). It has significant effects on fisheries, and in cases where it was removed from habitats, biodiversity and fish biomass massively increased ( Huseyinoglu et al., 2024). These aspects render the species an important ecosystem regulator; therefore, controlling its populations is of ecological and economic importance. Competition from the indigenous urchin species is forcing *D. setosum* to live on deeper bottoms, while its great size drives species to occupy open spaces, leaving rocks and small crevices free for the indigenous species, which are much smaller in size. The gonad size of the invasive urchin is also greater than that of the Mediterranean urchins. This attribute could favour *D. setosum* to dominate in common habitats.

Invasive *D. setosum* is spreading in the Eastern Mediterranean basin, and although mass mortality events have already been noted ( Skouradakis et al., 2024; Dinçtürk et al., 2024), the species attributes seem to support successful establishments. Moreover, overfishing of the indigenous urchin species benefits *D. setosum*, which is not exploited commercially. Therefore, describing a high-quality reference genome will expand our knowledge on traits, evolution, and adaptation of the Longspine Urchin and will contribute towards designing effective conservation strategies to control the species populations.

The generation of this reference resource was coordinated by the European Reference Genome Atlas (ERGA) initiative’s Biodiversity Genomics Europe (BGE) project, supporting ERGA’s aims of promoting transnational cooperation to promote advances in the application of genomics technologies to protect and restore biodiversity https://www.zotero.org/google-docs/?mS7euC ( Mazzoni et al., 2023; https://www.zotero.org/google-docs/?mS7euC).

## Materials & Methods

ERGA's sequencing strategy includes Oxford Nanopore Technology (ONT) and/or Pacific Biosciences (PacBio) for long-read sequencing, along with Hi-C sequencing for chromosomal architecture, Illumina Paired-End (PE) for polishing (i.e. recommended for ONT-only assemblies), and RNA sequencing for transcriptomic profiling, to facilitate genome assembly and annotation.

### Sample and sampling information

On 31st May 2023, a female adult of *Diadema setosum* was sampled and identified by Katerina Vasileiadou. The species identification through COI barcoding was confirmed by Katerina Vasileiadou. The specimen was collected by hand picking in Vlychia, Heraklion, Crete (Greece), under permission ΥΠΕΝ\ΔΔΔ\34284\1131 from the Ministry for Environment and Energy Secretariat General for Natural Environment & Water Directorate General for Forests & Forest Environment Directorate for Forest Management. The specimen's gonads were snap-frozen immediately after harvesting and stored in liquid nitrogen until DNA extraction.

### Vouchering information

Physical reference materials for the sequenced specimen were deposited in the Natural History Museum of Crete https://www.nhmc.uoc.gr/, under the accession number NHMC.65.24.

Frozen reference tissue material of the gonad is available from a proximal individual at the Biobank of the Natural History Museum of Crete https://www.nhmc.uoc.gr/, under the proxy voucher ID NHMC.65.24.

An electronic voucher image of the sequenced individual is available from ERGA’s EBI BioImageArchive dataset www.ebi.ac.uk/biostudies/bioimages/studies/S-BIAD1012?query=ERGA under accession ID https://ftp.ebi.ac.uk/biostudies/fire/S-BIAD/012/S-BIAD1012/Files/ERGA/SAMEA114349570_1.jpg.

### Data availability

*Diadema setosum* and the related genomic study were assigned to Tree of Life ID (ToLID) ‘eeDiaSeto1’, and all sample, sequence, and assembly information are available under the umbrella BioProject PRJEB77220 https://www.ebi.ac.uk/ena/browser/view/PRJEB77220. The sample information is available at the following BioSample accessions: SAMEA114349572, SAMEA114349578, SAMEA114349580. The genome assembly is accessible from ENA under accession number GCA_964275005.1.

Sequencing data produced as part of this project are available from ENA at the following accessions: ERX12733445, ERX12733463, ERX12737198, ERX12737199. Documentation related to the genome assembly and curation can be found in the ERGA Assembly Report (EAR) document available at https://github.com/ERGA-consortium/EARs/tree/main/Assembly_Reports/Diadema_setosum/eeDiaSeto1. Further details and data about the project are hosted on the ERGA portal at https://portal.erga-biodiversity.eu/data_portal/31175.

### Genetic information

The estimated genome size, based on ancestral taxa, is 1.17 Gb. This is a diploid genome with a haploid number of 22 chromosomes (2n = 11), and unknown sex chromosomes. All information for this species was retrieved from Genomes on a Tree https://www.zotero.org/google-docs/?wmNEAd ( Challis et al., 2023).

### DNA/RNA processing

DNA was extracted from 200 mg of gonads using a conventional CTAB extraction followed by a commercial purification using Qiagen Genomic tips (QIAGEN, MD, USA). A detailed protocol is available on protocols.io ( https://www.protocols.io/view/hmw-dna-extraction-for-long-read-sequencing-using-bp2l694yzlqe/v1). DNA fragment size selection was performed using Short Read Eliminator (PacBio, CA, USA). Quantification was performed using a Qubit dsDNA HS Assay kit (Thermo Fisher Scientific) and integrity was assessed in a FemtoPulse system (Agilent). DNA was stored at 4 ^o^C until usage.

RNA was extracted from gonads (50 mg) using the RNeasy Plus Universal kit (Qiagen) following the manufacturer's instructions. Residual genomic DNA was removed with 6U of TURBO DNase (2 U/μL) (Thermo Fisher Scientific). Quantification was performed using a Qubit RNA HS Assay kit, and integrity was assessed in a Bioanalyzer system (Agilent). RNA was stored at -80 °C.

### Library preparation and sequencing

Long-read DNA libraries were prepared with the SMRTbell prep kit 3.0 following manufacturers' instructions and sequenced on a Revio system (PacBio).

Hi-C libraries were generated from gonads (50 mg) of the same individual using the Arima High Coverage HiC kit (following the Animal Tissues low input protocol v01) and sequenced on a NovaSeq6000 instrument (Illumina) with 2x150 bp read length.

Poly(A) RNA-Seq libraries were constructed using the Illumina Stranded mRNA Prep, Ligation Prep kit (Illumina) and sequenced on the Illumina NovaSeq6000 instrument (Illumina) with 2x150 bp read length.

### Genome assembly methods

The genome of *Diadema setosum* was assembled using the Genoscope GALOP pipeline ( https://workflowhub.eu/workflows/1200). Briefly, raw PacBio HiFi reads were assembled using Hifiasm v0.19.5-r593. Retained haplotigs were removed using purge_dups v1.2.5 with default parameters and the proposed cutoffs. The purged assembly was scaffolded using YaHS v1.2, and assembled scaffolds were then curated through manual inspection using PretextView v0.2.5 to remove false joins and incorporate sequences not automatically scaffolded into their respective locations within the chromosomal pseudomolecules.

Chromosome-scale scaffolds confirmed by Hi-C data were named in order of size. The mitochondrial genome was assembled using Oatk v1.0 and included in the released assembly. Summary analysis of the released assembly was performed using the ERGA-BGE Genome Report ASM Galaxy workflow ( https://doi.org/10.48546/workflowhub.workflow.1104.1).

## Results

### Genome assembly

**Figure 1.**
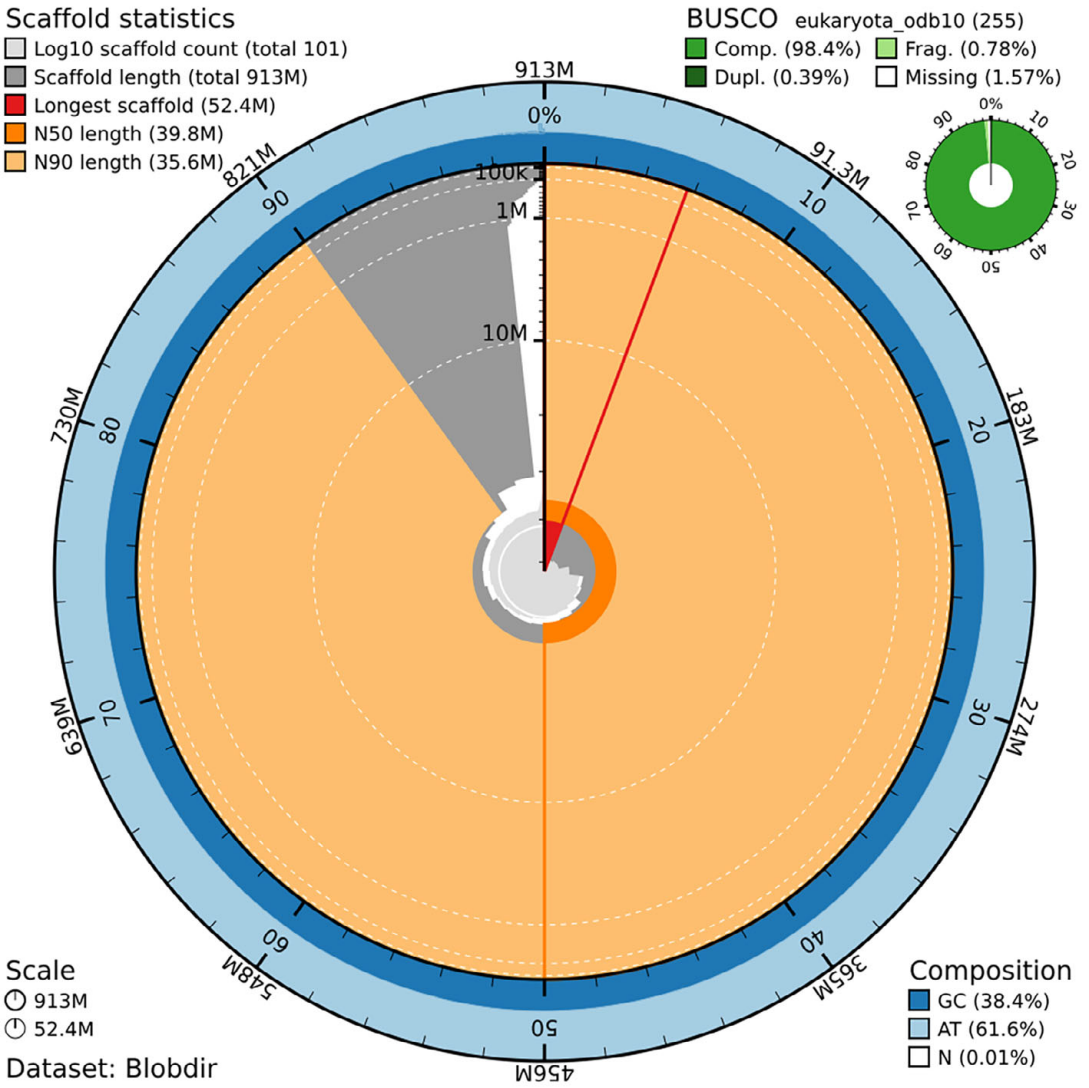
Snail plot summary of assembly statistics. The main plot is divided into 1,000 size-ordered bins around the circumference, with each bin representing 0.1% of the 912,728,646 bp assembly, including the mitochondrial genome. The distribution of sequence lengths is shown in dark grey, with the plot radius scaled to the longest sequence present in the assembly (52.4 Mb, shown in red). Orange and pale-orange arcs show the scaffold N50 and N90 sequence lengths (39.8 Mb and 35.6 Mb), respectively. The pale grey spiral shows the cumulative sequence count on a log-scale, with white scale lines showing successive orders of magnitude. The blue and pale-blue area around the outside of the plot shows the distribution of GC, AT, and N percentages in the same bins as the inner plot. A summary of complete, fragmented, duplicated, and missing BUSCO genes found in the assembled genome from the Eukaryota database (odb10) is shown on the top right.

**Figure 2.**
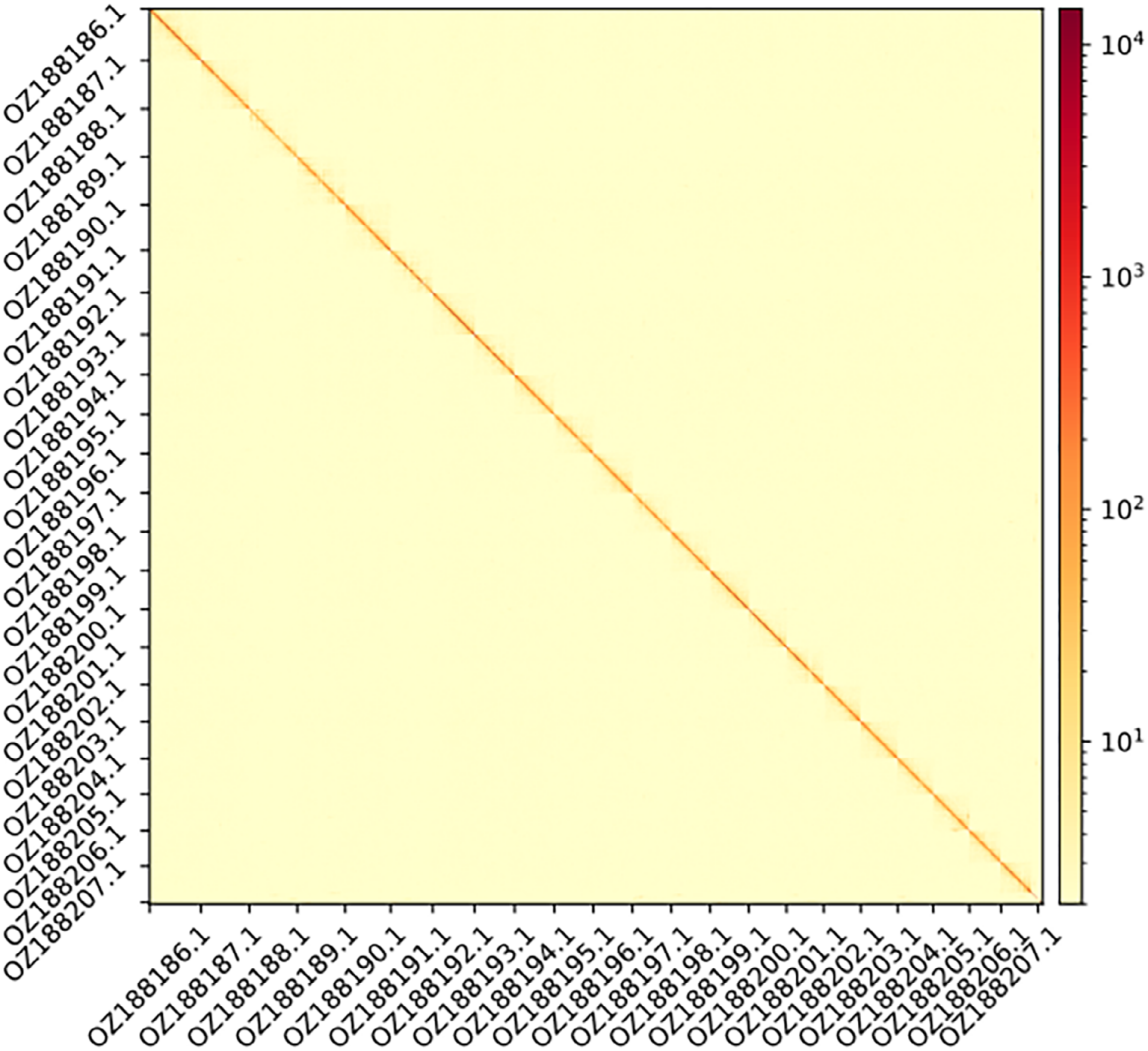
Hi-C contact map showing spatial interactions between regions of the genome. The diagonal corresponds to intra-chromosomal contacts, depicting chromosome boundaries. The frequency of contacts is shown on a logarithmic heatmap scale. Hi-C matrix bins were merged into a 150 kb bin size for plotting.

The genome assembly has a total length of 912,728,646 bp in 101 scaffolds, including the mitogenome ( Figures 1 and 2), with a GC content of 38.35%. It features a contig N50 of 2,178,457 bp (L50 = 133) and a scaffold N50 of 39,828,611 bp (L50 = 11). There are 644 gaps, totaling 66,200 kb in cumulative size. The single-copy gene content analysis using the Eukaryota database with BUSCO (Manni et al., 2021) resulted in 98.4% completeness (98.0% single and 0.4% duplicated). 76.9% of reads k-mers were present in the assembly, and the assembly has a base accuracy Quality Value (QV) of 55.6% as calculated by Merqury https://www.zotero.org/google-docs/?8tN3un ( Rhie et al., 2020).

## Author contributions

KV and TM coordinated the project, KV collected the species, identified the species, sampled and preserved biological material and provided metadata, RM, AsB, RF and NE provided sampling and metadata support and management, GST extracted DNA, prepared libraries, and performed sequencing under the supervision of AM, CC, KL and PHO; LD, AC, BI and JMA performed genome assembly and curation under the supervision of JMA; RM generated the analysis and report. All authors contributed to the writing, review, and editing of this genome note and read and approved the final version.

## Author information

Members of the Genoscope Sequencing Team are listed here: https://doi.org/10.5281/zenodo.14611490.
