# Supplementary material for: ERGA-BGE reference genome of Diadema setosum: the Black Longspine Urchin invading the Mediterranean sea
Source: Open Res Eur. 2026 Mar 12;6:75. [Version 1] doi: 10.12688/openreseurope.20681.1 (PMC13264686; doi:10.12688/openreseurope.20681.1)
Supplement: Peer review history

# Reviewer report: Reviewer response for version 1

**DOI:** 10.21956/openreseurope.22371.r75610

**Published:** 2026-06-13

**Authors:** Qinggang Xue

**Affiliations:** 1 Zhejiang Wanli University, Ningbo, Zhejiang, China

This high-quality chromosome-level genome assembly of the invasive *Diadema setosum* fills a critical gap in understanding Mediterranean Lessepsian migrant adaptation. With strong BUSCO completeness and EBP-aligned metrics, it supports conservation efforts—minor clarifications on assembly details will enhance its utility. Recommended for Indexing

 Reviewer Comments

1. The manuscript states a diploid chromosome number of 2n=11 but later reports 22 chromosomal pseudomolecules. Resolve this contradiction (likely a typo; should be 2n=22) and confirm with cytogenetic evidence or ancestral taxon comparisons.
2. The Materials and Methods do not specify key parameters for the GALOP pipeline (e.g., Hifiasm assembly settings, purge_dups cutoff values). Supplement with detailed parameters to ensure reproducibility of the assembly workflow.
3. The RNA-seq data are mentioned for transcriptomic profiling but not utilized for genome annotation in this note. Clarify when the annotated genome will be released and whether preliminary analyses of adaptive genes (e.g., temperature tolerance, grazing-related loci) were conducted.
4. The Results report 76.9% k-mer completeness, which is lower than typical standards for reference genomes. Discuss potential reasons (e.g., repeat content, heterozygosity) and how this impacts the assembly’s utility for downstream analyses.
5. The study focuses on a single female specimen—note whether sex-specific genomic features (e.g., sex chromosomes) were investigated and if sampling a single individual limits insights into population-level adaptive variation.
6. The Hi-C contact map (Figure 2) lacks a clear legend for the logarithmic heatmap scale and chromosome labeling. Revise the figure to include these details and improve readability.
7. The Introduction highlights *D. setosum*’s ecological impacts but does not compare its genome to native Mediterranean urchin species. Add a brief comparative context to emphasize unique adaptive traits of the invader.

Are sufficient details of methods and materials provided to allow replication by others?

Partly

Is the rationale for creating the dataset(s) clearly described?

Yes

Are the datasets clearly presented in a useable and accessible format?

Yes

Are the protocols appropriate and is the work technically sound?

Yes

Reviewer Expertise:

marine biology, genetics, evolution

I confirm that I have read this submission and believe that I have an appropriate level of expertise to confirm that it is of an acceptable scientific standard.

# Reviewer report: Reviewer response for version 1

**DOI:** 10.21956/openreseurope.22371.r71383

**Published:** 2026-04-08

**Authors:** Bingpeng Xing

**Affiliations:** 1 Third Institute of Oceanography Ministry of Natural Resources, Xiamen, China

The manuscript reports a chromosome-level reference genome for Diadema setosum. As an important invasive sea urchin in the Mediterranean, this species has clear ecological relevance, so the genomic resource itself is valuable. The authors used PacBio HiFi and Hi-C for genome assembly, which is an appropriate strategy for generating a high-quality reference genome.

 The main issue at present is that several key methodological details are missing or described too briefly, making the assembly workflow insufficiently clear and difficult to reproduce. For example, the manuscript mentions ONT, PacBio, Illumina, and RNA-seq in the overall sequencing strategy, but it does not clarify whether ONT data were actually used for this species, and if so, at which step. Although hifiasm is listed with its version, key parameters are not provided. The description of purge_dups is also too vague, as the actual cutoff values are not reported. I suggest that the authors describe the assembly workflow more clearly, step by step, and provide the relevant software versions and key parameter settings.

 A second major issue is that RNA-seq and annotation are mentioned in the Methods, but no corresponding annotation results are presented in the Results. If genome annotation was indeed carried out, the manuscript should at least report the number of predicted genes and provide a brief summary of the annotation. If annotation is not part of the formal scope of this paper, then the wording in the Methods should be revised accordingly. As written, the manuscript gives the impression that annotation was completed, but no results are actually shown.

 In addition, there are inconsistencies in the genome quality assessment. Different BUSCO datasets and versions are mentioned in different parts of the manuscript, including Metazoa_odb12, Eukaryota, and odb10, and these should be made consistent throughout. Another concern is that the reported genome size of 1.17 Gb appears to come from previously available database information rather than from an independent estimate based on the present sample. Given that the final assembly size is only about 0.91 Gb, I recommend adding a k-mer–based estimate of genome size, heterozygosity, and repeat content, and briefly discussing the discrepancy.

 Contamination assessment is also a relatively weak point in the current manuscript. It is not clearly stated whether contamination screening was performed, how potentially contaminant contigs were identified, or whether any contigs were removed.

 In addition, the manuscript states that the mitochondrial genome was assembled and included in the final release, but no basic information is provided in the main text. At minimum, the authors should report the mitochondrial genome size, gene content, and annotation method.

 There are also a few issues of accuracy and presentation that should be corrected. For example, the statement “a diploid genome with a haploid number of 22 chromosomes (2n = 11)” is biologically incorrect and should be revised. The Merqury result should also be written as QV 55.6 rather than 55.6%. More generally, the manuscript would benefit from a careful check of wording and formatting to ensure consistency in terminology, software/database versions, and reference formatting throughout.

Are sufficient details of methods and materials provided to allow replication by others?

Partly

Is the rationale for creating the dataset(s) clearly described?

Yes

Are the datasets clearly presented in a useable and accessible format?

Partly

Are the protocols appropriate and is the work technically sound?

Partly

Reviewer Expertise:

genome，marine biology，DNA，phylogenetic analysis

I confirm that I have read this submission and believe that I have an appropriate level of expertise to confirm that it is of an acceptable scientific standard, however I have significant reservations, as outlined above.

# Reviewer report: Reviewer response for version 1

**DOI:** 10.21956/openreseurope.22371.r71388

**Published:** 2026-03-30

**Authors:** Abdelmalek Lekired

**Affiliations:** 1 University of New Mexico, Albuquerque, New Mexico, USA

**Reviewer Report**

 I would like to thank the authors for submitting their manuscript entitled *“Reference genome of Diadema setosum: the Black Longspine Urchin invading the Mediterranean Sea.”* The generation of a chromosome-level genome for this ecologically important and invasive species represents a valuable contribution to the field. The use of HiFi long reads combined with Hi-C scaffolding is appropriate and has strong potential to produce a high-quality reference genome.

 However, several methodological details are currently missing, and some analyses require clarification or improvement to meet current standards for genome assembly and annotation studies. I outline below my major comments and suggestions.

**Abstract**

 The abstract would benefit from including key assembly statistics to better reflect the quality of the genome. I recommend adding:

- Genome size
- Contig/scaffold N50
- BUSCO completeness scores

Including these metrics will provide readers with a clearer overview of the assembly quality.

**Materials and Methods**

 The authors state that ONT, PacBio, Illumina, and RNA-seq data were used for assembly and annotation. However, several important details are missing:

1. The role of ONT reads in the assembly is not described. It is unclear whether they were used for scaffolding, gap filling, polishing, or not used at all. This must be clarified.
2. The number of RNA-seq samples, their origin (tissues, developmental stages), and sequencing depth are not provided. These details are essential for evaluating the annotation quality.
3. Annotation methods are mentioned, but no corresponding results are presented. Either the annotation results should be included, or the annotation section should be removed or revised.
4. The authors mention using the BUSCO dataset *Metazoa_odb12*, but the results are not reported anywhere in the manuscript. This inconsistency must be addressed.

**General Concerns**

1. The genome size is reported as 1.17 Gb based on previously published data. The authors should estimate the genome size independently using k-mer–based approaches (e.g., GenomeScope2) and report heterozygosity and repeat content.
2. The statement:
*“This is a diploid genome with a haploid number of 22 chromosomes (2n = 11)”*
  is incorrect and biologically inconsistent. If 2n = 22, then n = 11. This should be corrected.

**Genome Assembly Methods**

 This section lacks sufficient detail and is currently not reproducible.

- The authors mention the use of ONT reads but do not describe how they were incorporated into the assembly workflow.
- If *hifiasm* was used, relevant parameters should be reported (e.g., options related to duplicate purging such as -l 0 or -l 3, if applicable).
- The statement:
*“Retained haplotigs were removed using purge_dups v1.2.5 with default parameters and the proposed cutoffs”*
  is unclear. The authors should clearly define what is meant by “retained haplotigs” and specify the cutoff values used.

Overall, the assembly pipeline should be described step-by-step with software versions and parameters to ensure reproducibility.

**Results**

 Several inconsistencies and missing details need to be addressed:

1. Two BUSCO versions ( *odb10* and *odb12*) are mentioned. The authors should clarify which dataset was used and ensure consistency throughout the manuscript.
2. The results should include BUSCO evaluation using an appropriate lineage dataset (preferably Metazoa). If a eukaryotic dataset was used, the rationale should be explained.
3. The manuscript does not address potential contamination:
  - Were contaminants assessed (e.g., using BlobToolKit or similar tools)?
  - If so, how were contaminant contigs identified and removed?
4. Regarding the blob plot:
  - The dataset labeling should reflect the genome name rather than generic directory names (e.g., “BlobDir”).
  - The figure and legend should be revised for clarity.
5. The snail plot:
  - It should be clearly stated whether this plot corresponds to the final chromosome-level assembly.
6. The mitochondrial genome assembly is mentioned, but no details are provided. The authors should report (if available):
  - Genome size
  - Number of protein-coding genes
  - rRNA and tRNA content
  - Annotation approach used

**Conclusion**

 This study presents a valuable genomic resource for *Diadema setosum*, but the manuscript requires **substantial revision** to improve clarity, reproducibility, and completeness. Addressing the issues outlined above will significantly strengthen the manuscript and bring it in line with current standards for genome assembly and annotation studies.

Are sufficient details of methods and materials provided to allow replication by others?

No

Is the rationale for creating the dataset(s) clearly described?

Yes

Are the datasets clearly presented in a useable and accessible format?

Yes

Are the protocols appropriate and is the work technically sound?

Yes

Reviewer Expertise:

Computational genomics and bioinformatics, with specialization in de novo genome assembly (long-read and illumina short reads), genome curation, gene prediction and functional annotation, transcriptome assembly, RNA-Seq analysis, ploidy inference, and genome quality assessment (e.g., BUSCO, k-mer analysis). My research primarily focuses on non-model organisms, including metazoan genomes.

I confirm that I have read this submission and believe that I have an appropriate level of expertise to confirm that it is of an acceptable scientific standard, however I have significant reservations, as outlined above.

## References

[1] : Whole-genome sequencing and genome-wide transcriptome profiling of the freshwater planorbid snail Bulinus ugandae (Mollusca: Gastropoda), a Nilotic bulinine refractory to Schistosoma haematobium. *BMC Genomics* .2025;26(1) : 10.1186/s12864-025-12320-3 10.1186/s12864-025-12320-3 41361373 PMC12690972
